# Recombinant Human Tissue Kallikrein-1 for Treating Acute Ischemic Stroke and Preventing Recurrence

**DOI:** 10.1161/STROKEAHA.124.048858

**Published:** 2025-01-17

**Authors:** Scott E. Kasner, Philip M. Bath, Michael D. Hill, John J. Volpi, Michael Giuffre, Lorianne Masuoka, David Wambeke, Paolo R. Madeddu

**Affiliations:** University of Pennsylvania School of Medicine, Division of Vascular Neurology, Philadelphia (S.E.K.).; Stroke Trials Unit, University of Nottingham, United Kingdom (P.M.B.).; Department of Clinical Neuroscience and Hotchkiss Brain Institute, Cumming School of Medicine, University of Calgary and Foothills Medical Centre, Alberta, Canada (M.D.H.).; Houston Methodist, Department of Neurology, Houston, TX (J.J.V.).; Faculty of Medicine, University of Calgary, Alberta, Canada (M.G.).; DiaMedica Therapeutics, Minnetonka, MN (L.M., D.W.).; Experimental Cardiovascular Medicine, University of Bristol, United Kingdom (P.R.M.).

**Keywords:** bradykinin, cerebral infarction, kallikreins, stroke, vasodilation

## Abstract

Novel strategies are needed for the treatment of acute ischemic stroke when revascularization therapies are not clinically appropriate or are unsuccessful. rKLK1 (recombinant human tissue kallikrein-1), a bradykinin-producing enzyme, offers a promising potential solution. In animal studies of acute stroke, there is a marked 36-fold increase in bradykinin B2 receptor on brain endothelial cells of the ischemic region. Due to this environment, rKLK1-generated bradykinin will exert a potent local vasodilation and increase brain perfusion via 3 synergistic signaling pathways downstream to the B2 receptor. Because of its preferential effect on ischemic tissue, systemic adverse effects such as hypotension are avoided with proper dosing. In addition, with initial vasodilation through recruitment of preexisting collaterals, rKLK1 promotes long-term benefit of brain perfusion by promoting new collateral formation. With an extended course of therapy for weeks after acute ischemic stroke, these multifaceted effects may also reduce the risk of stroke recurrence. A prior phase II trial demonstrated a favorable impact on clinical outcomes and recurrent strokes, particularly among patients who were not eligible for mechanical thrombectomy. A phase II/III trial has launched in this population, though opportunities for combination revascularization therapies deserve further investigation.

Acute ischemic stroke (AIS) is a leading cause of death and disability, resulting in an estimated global health care cost of $721 billion annually.^1^ Acute treatment options have been predominantly limited to reperfusion therapies with intravenous thrombolysis (IVT) and mechanical endovascular thrombectomy (EVT). Remarkably, there has been no new pharmaceutical agent widely adopted worldwide for AIS in over 25 years. Moreover, IVT and EVT are out of reach for many patients due to multiple selection factors, most prominently the narrow therapeutic time window for IVT and the absence of a large vessel occlusion or limited access to centers with this EVT capability.

Several investigational therapies have been tested but have not consistently shown efficacy, although some have been approved in certain countries based on limited data. One such novel therapy is Kailikang, a semipurified form of human KLK1 (tissue kallikrein-1) derived from human urine. Kailikang has been tested as a treatment in thousands of patients with AIS and has received approval for this indication in China. KLK1 works by enhancing perfusion in the ischemic brain through the generation of bradykinin, a peptide that induces vasorelaxation in vasculature. Additionally, KLK1 promotes angiogenesis, taking a few days to form new collateral vessels at the ischemic site. The acute and long-term effects of KLK1 both contribute to maintaining the viability of brain vascular cells in the stroke penumbra. Unlike neuroprotectants that focus on enhancing brain resilience, KLK1 specifically targets the improvement of regional blood flow to ischemic areas, in a mechanism that is distinct from and potentially complementary to recanalization treatments.

The urinary origin of Kailikang poses significant challenges for regulatory approval and patient acceptance. Rinvecalinase alfa (also known as DM199) is an rKLK1 (recombinant KLK1) with a molecular structure similar to endogenous KLK1 and Kailikang. Rinvecalinase alfa represents a promising newer approach to AIS that noninvasively enhances early reperfusion by preferentially dilating collateral arteries in the ischemic penumbra, with the potential for sustained treatment beyond the acute period via formation of new collaterals. Notably, with a 24-hour treatment window, rKLK1 can potentially serve the many patients with AIS ineligible for IVT or EVT with a low risk for hemorrhagic complications.^2^

## Critical Role of Collateral Circulation in Ischemic Stroke Recovery

During AIS, decreased perfusion pressure distal to the occluded vessel creates a pressure gradient, which enhances blood flow through preexisting collateral vessels. This compensatory response, in association with collateral vasorelaxation (a process called collateral recruitment), is critical for maintaining perfusion of the penumbra and may also facilitate spontaneous thrombus dissolution by the endogenous fibrinolytic system.^3^ Effective recruitment of collateral vessels correlates with a higher Alberta Stroke Program Early CT Score, indicating a smaller presumed infarct core at baseline, a slower rate of infarct progression, and a smaller final infarct volume.

Unfortunately, risk factors such as aging, hypertension, atherosclerosis, and diabetes can weaken spontaneous collateral recruitment, making it inefficient in supporting the full recovery of the at-risk area. Additionally, common anatomic variations in the circle of Willis and secondary vessels can limit the development and functionality of collateral circulation, further hindering recovery.^4–6^ To further optimize cerebral perfusion, permissive hypertension, or the practice of tolerating a high blood pressure after AIS, is sometimes used in the first few days. However, this approach may not enhance focal cerebral blood flow. It must be carefully managed to prevent cerebral hemorrhage or edema or other end-organ damage, such as to the heart or kidneys.

Collateral circulation is essential for all patients experiencing AIS and is especially crucial for those not successfully revascularized. In the absence of recanalization, collateral circulation must be maintained until the thrombus naturally dissolves, a process that may take days or weeks. Moreover, some patients may also experience the no-reflow phenomenon, where distal microvascular (tissue-level) blood flow remains impaired despite therapeutic or spontaneous recanalization. Currently, no pharmaceutical agents are available that can immediately and sustainably enhance collateral circulation after an AIS.

## KLK1 and Endothelial Kinin B2 Receptor Activation: Key Drivers in the Production of Nitric Oxide, Prostacyclin, and Endothelium-Derived Hyperpolarizing Factor

**Figure 1.**
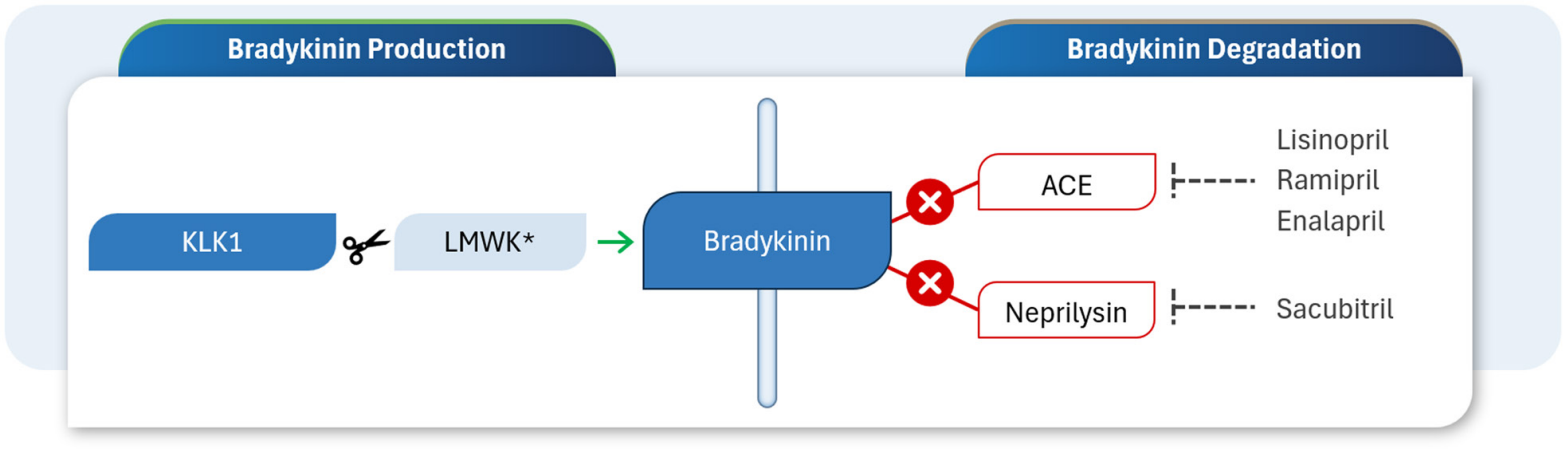
**Pathways of bradykinin production and degradation using KLK1 (tissue kallikrein-1).** KLK1 cleaves LMWK to generate bradykinin. Angiotensin-converting enzyme (ACE) and neprilysin cleave bradykinin and render it inactive. *Low-molecular-weight kininogen.

KLK1 is a serine proteinase with a molecular weight of ≈38 kDa. Kidneys, pancreas, and lungs, as well as the vasculature and brain, are major sources of KLK1. The protein is also secreted in biological fluids (urine, pancreatic, and salivary gland fluid) and reaches distant organs through the bloodstream. The primary role of KLK1 is to perform proteolytic cleavage of low-molecular-weight kininogen to make the kinin peptides, bradykinin, and lys-bradykinin (collectively referred to as bradykinins or BK; Figure 1). KLK1 is the main bradykinin-producing enzyme during resting conditions, while angiotensin-converting enzyme is the main bradykinin-degrading enzyme. One benefit of angiotensin-converting enzyme inhibitors, commonly prescribed as first-line therapies for arterial hypertension, is that they enhance the effects of endogenous bradykinin by inhibiting its degradation. However, they do not create new bradykinin. Similarly, sacubitril (a component of Entresto) is used to treat heart failure, acting as an inhibitor of neprilysin, another bradykinin-degrading enzyme.

KLK1 and plasma kallikrein are distinct in their molecular structures. While both can produce bradykinin, KLK1 cleaves low-molecular-weight kininogen, whereas plasma kallikrein cleaves high-molecular-weight kininogen. Importantly, KLK1 does not cleave plasminogen to form plasmin, unlike plasma kallikrein. Plasmin is involved in the fibrinolytic, complement, and contact systems.^7^

Bradykinin acts as a cell signaling molecule upon engagement with the B1 (bradykinin 1) and B2 (bradykinin 2) receptors. Age-related increases in B1 and B2 receptor levels that have been previously reported in mice cardiac and human cardiac tissue.^8,9^ Mice brain tissue studies have shown that the expression of kinin B1 and B2 receptors changes with age, with younger mice having higher levels of both receptors, especially B2, while older mice exhibit increased B2 receptor expression only when B1 receptors are blocked and generally lower B1 receptor levels.^10^ The B1 receptor is inducible, typically synthesized in pathological situations, whereas the B2 receptor is constitutively expressed, that is, continually synthesized in the cardiovascular system. Receptor binding by BK stimulates many cellular cascades, causing vascular smooth cells in arterioles to relax. This relaxation is mediated in part by the release of nitric oxide (NO) and prostacyclin (PGI2) from the endothelium. NO activates soluble guanylate cyclase to increase the production of cyclic GMP (cGMP), facilitating vasodilation in a paracrine manner. Similarly, PGI2 elevates cyclic AMP (cAMP) levels, enhancing the relaxation effects and contributing to the vasodilatory response in neighboring vascular smooth cells. A third cellular cascade includes the endothelium-derived hyperpolarizing factor, which induces vasorelaxation even when normal NO- and PGI2-mediated signaling pathways are impaired. According to the site of production, the KLK system exerts specialized functions. For instance, in kidneys, the system exerts diuretic and natriuretic functions, while in the vasculature it acts as a relaxant and healing mechanism.^11^ Of particular interest is the emerging role of the KLK1 system in maintaining homeostasis of the brain vasculature.^12,13^

KLK1 levels have been reported to be reduced in patients with cardiovascular and renal disease.^6^ Low KLK1 levels are also associated with increased risk for first-ever stroke, stroke recurrence, and stroke mortality.^14^ As a consequence of the endogenous KLK1 deficit, the production of vasodilating BK will be reduced, although partially compensated by a reported upregulation of kinin B2 receptor in the ischemic brain hemisphere.^15–17^ KLK1 augmentation therapy could enhance cerebral perfusion through increased BK production.

## Facilitating Focal Vasodilation: BK2 Receptor Upregulation and KLK1’s Role in Collateral Circulation

**Figure 2.**
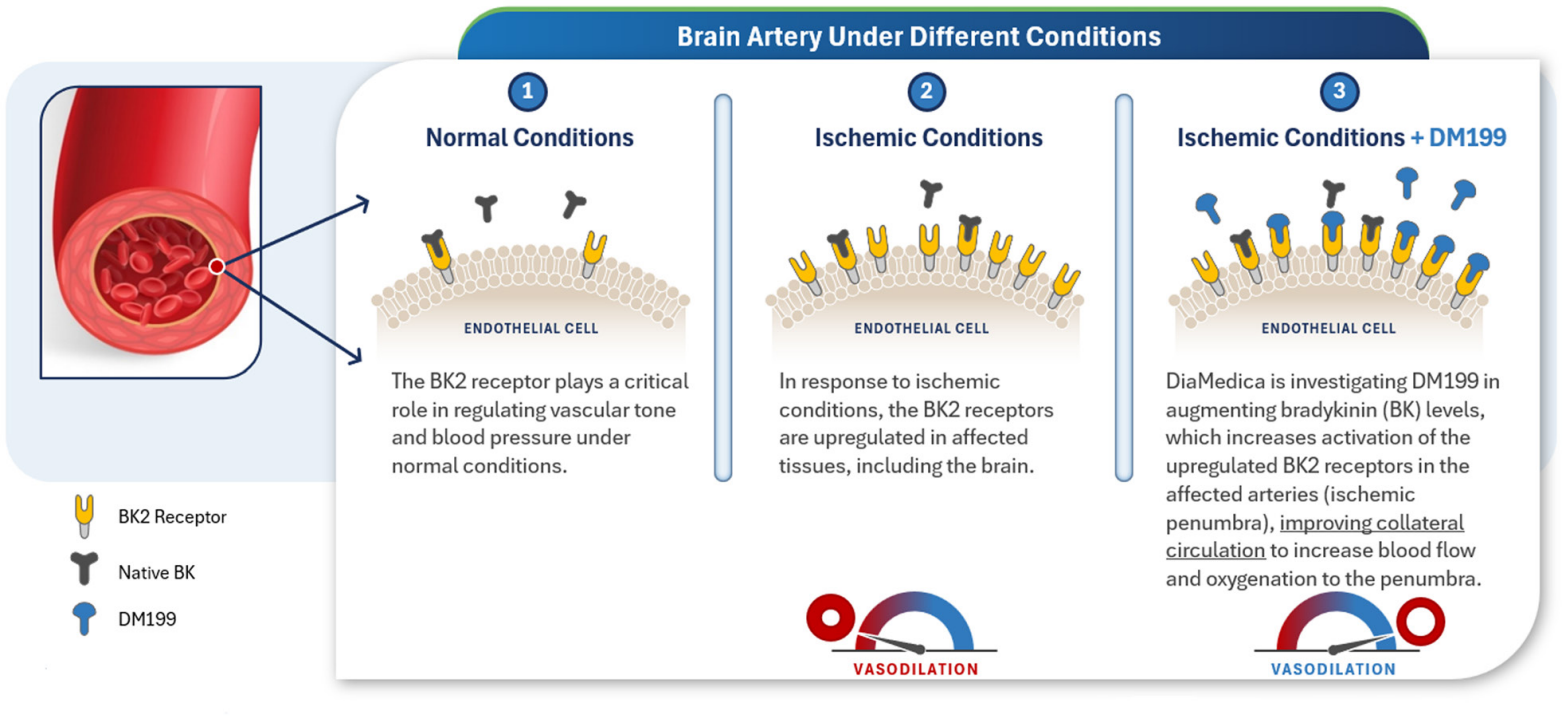
Brain ischemia induces the upregulation of bradykinin B2 receptors with consequent enhanced vasodilation after KLK1 (tissue kallikrein-1)-induced bradykinin release.

Traditional vasodilators have generally been ineffective in stroke treatment, likely due to their systemic effects. Vasodilatory medications typically lower systemic blood pressure, which counterproductively decreases the blood supply to the brain during ischemic conditions, undermining the intended benefits of controlled permissive hypertension. Recombinant KLK1 may resolve this paradox by inducing focal vasodilation in the ischemic penumbra. In animal models of AIS, the expression of the BK2 receptor in the endothelial cells was found to increase 36-fold in the ipsilateral middle cerebral artery, compared with a contralateral increase of 10-fold.^16^ Thus, the binding of KLK1 produces bradykinin, and in AIS, it capitalizes on the upregulated BK2 receptors for improving brain perfusion under ischemic conditions by enhancing focal vasodilation in the ischemic penumbra and augmenting local cerebral blood flow without significantly affecting systemic blood pressure. With sustained therapy for weeks after the acute injury, this approach is expected to minimize infarct size and reduce the severity of stroke and also enhance collateral circulation, potentially reducing the risk of early stroke recurrence (Figure 2).

**Figure 3.**
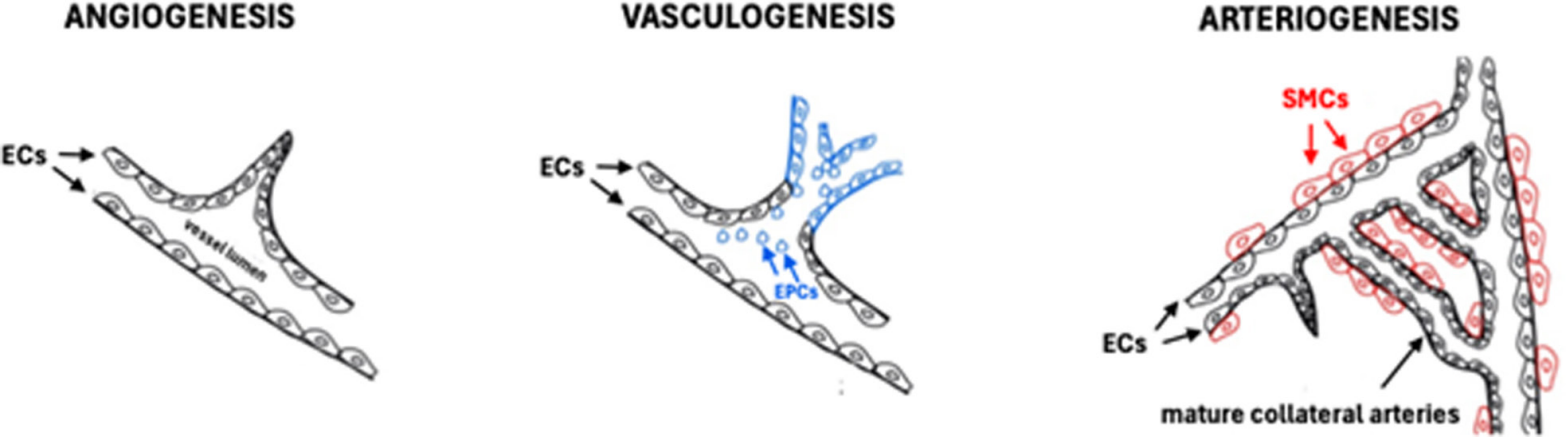
**Mechanisms of angiogenesis, vasculogenesis, and arteriogenesis.** EC indicates endothelial cell; and SMC, smooth muscle cell. Reprinted from Szöke et al^20^ with permission. Copyright ©2012, Oxford University Press.

Collateral vessel recruitment after AIS is rapid, occurring on the order of seconds in rodent studies.^16^ In addition to collateral recruitment, administering KLK1 reportedly improved long-term perfusion by enhancing the spontaneous processes of collateral remodeling and new collateral formation, as evidenced in experimental AIS models.^17–19^ The collateralization process encompasses 3 distinct but mutually synergic mechanisms: angiogenesis, vasculogenesis, and arteriogenesis. Angiogenesis, the growth of blood vessels from existing blood vessels, is a reparative mechanism that starts with the induction of endothelial cell proliferation and migration, leading to new capillary sprouting from preexisting blood vessels. Vasculogenesis, the process of creating new blood vessels based on the absence of existing blood vessels, relies on endothelial progenitor cells that are attracted from the circulation to the ischemic brain after a cytokine gradient. The maturation of capillaries generated through angiogenesis and vasculogenesis occurs via arteriogenesis, which consists of capillaries being covered by smooth muscle cells and pericytes. De novo formation of new arterioles can also occur through the formation of bridges between arterioles (Figure 3).^20^

In a mouse model, KLK1 treatment increased the density of capillary endothelial cells as early as 1 day after cerebral ischemia,^18^ and activation of arteriogenesis has been found through angiography and other imaging techniques after AIS in patients.^21^ KLK1-produced bradykinins promote arteriogenesis by attracting and recruiting mural vascular cells, such as pericytes and smooth muscle cells, that wrap around capillaries.^22–28^ Moreover, bradykinin attracts pro-angiogenic and pro-arteriogenic mononuclear cells that express the BK2 receptors from circulation.^29^ Next, the fusion of these new arterioles to other vessels generates new vessel anastomoses.^30^ At a molecular level, bradykinin-induced neovascularization (a single term used to encompass angiogenesis, vasculogenesis, and arteriogenesis) involves the activation of both the BK1 and BK2 receptors.^18,23,27,31^ Ultimately, this cascade releases several molecules, such as NO, VEGF (vascular endothelial growth factor), and apelin.^18^

## KLK1 Therapy Surpasses Other Collateral Enhancement Attempts

Clinical trials have tested other interventions to improve collateral vessel enhancement. Despite promising preclinical studies, the results of interventions such as remote ischemic postconditioning, sphenopalatine ganglion stimulation, mild induced hypertension, or statins have been disappointing or inconclusive.^32–35^ In experimental animal studies, one showed that inhaled NO induced selective vasodilation in the ischemic penumbra and improved penumbral metabolism.^36^ However, early administration of transdermal nitroglycerin, which is converted to NO in endothelial cells, failed to improve the outcomes of AIS participants in the recent phase III clinical trials RIGHT-2 (Rapid Intervention With Glyceryl Trinitrate in Hypertensive Stroke Trial-2) and MR ASAP (Multicenter Randomized Trial of Acute Stroke Treatment in the Ambulance With a Nitroglycerin Patch).^37,38^

In a longer-term context, the LACI-2 randomized clinical trial (Lacunar Intervention Trial-2) used escalating doses of isosorbide mononitrate, a NO donor, and cilostazol, a phosphodiesterase enzyme inhibitor, which augments the prostacyclin-cAMP pathway. Additionally, the trial explored a combination of these 2 agents to assess synergistic effects. The combination of these 2 interventions started months after the lacunar stroke and administered over a year significantly reducing the risk of major vascular events and improving functional and cognitive outcomes compared with single-agent administration, with a good safety profile.^39^ These data support the premise that prolonged targeting of multiple vasorelaxant pathways via NO and prostacyclin may serve to reduce subsequent risk of stroke recurrence and improve functional stroke recovery.

Notably, by activating the kinin BK2 receptor signaling in the ischemic brain vasculature, KLK1 stimulates 3 different vasoactive pathways at once: NO, prostacyclin, and endothelium-derived hyperpolarizing factor, thereby ensuring a maximal and coordinated response even in conditions of endothelial dysfunction.^40^ In addition, activation of the BK2 receptor has been shown in animal models to transactivate the VEGF receptor, which stimulates angiogenesis and cellular repair mechanisms.^41^ An extended course of therapy for weeks after the AIS is expected to support and sustain these effects.

## Previous Trials Using KLK1 Extracted From Human Urine Demonstrate Improved Perfusion and Favorable Outcomes in AIS

Human urinary kallikrein (HUK) is a natural form of KLK1 and is trademarked as Kailikang. This agent is approved in China for treating AIS, with evidence of benefits on neurological indices and perfusion measures. It is approved for use up to 48 hours after stroke onset, providing a significant extension beyond the 4.5-hour limit for IVT. Based on publicly disclosed sales figures, over 500 000 patients are estimated to be treated annually with HUK for AIS.^12^

**Figure 4.**
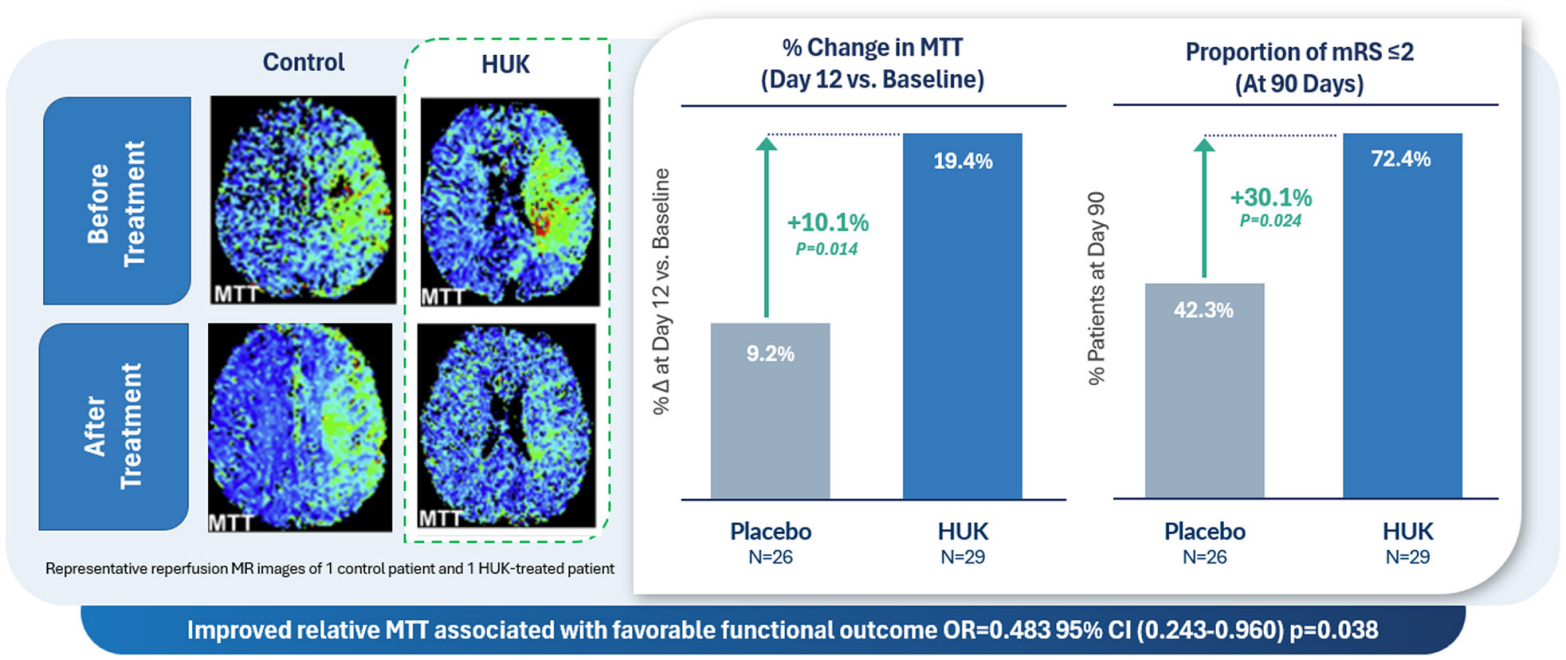
**Improved relative perfusion (mean transit time [MTT]) associated with favorable functional outcome (odds ratio [OR], 0.483 [95% CI, 0.243–0.960]; *P*=0.038).** HUK indicates human urinary kallikrein; and MR, magnetic resonance. Adapted from Li et al^42^ with permission. Copyright ©2015, Elsevier.

Most HUK trials in China have used a standard dosing protocol of 1 daily IV infusion of HUK for 14 to 21 days. In 1 small randomized trial of 58 patients with AIS (Figure 4), treatment with HUK reduced ischemic penumbra and reduced infarct size on magnetic resonance imaging obtained at 12 days and was associated with better functional outcomes as measured by the modified Rankin Scale score at 90 days poststroke.^42^

In another trial of 30 patients, HUK administration improved cerebral blood flow in the ischemic hemisphere without affecting blood flow in the contralateral hemisphere.^43^ The experimental group, consisting of 18 participants, was treated with intravenous HUK (0.15 units/d) for 7 consecutive days. The control group was given routine medication. Compared with baseline, HUK treatment was associated with improved blood flow. Moreover, additional perfusion indexes were improved in the HUK-treated group compared with the placebo group.^43^ In the DM199 ReMEDy1 phase 2 AIS trial (Randomized, Double‐Blind, Placebo‐Controlled Phase II Multi‐CenterEvaluation to Assess the Safety and Tolerability of DM199 Administered Intravenously andSubcutaneously in Subjects With Acute Ischemic Stroke), among patients not treated with mechanical thrombectomy, 24% of rKLK1 patients compared with 9.5% placebo had modified Rankin Scale score of 0 to 1 at 90 days (N=46).^44^

Numerous clinical studies have reported that treatment with HUK was safe, and the therapeutic action persisted with time and led to a reduction of AIS recurrence.^2,45–47^ For example, in the 2021 RESK study (Reevaluate the Efficacy and Safety of Human Urinary Kallidinogenase), which enrolled over 1200 participants undergoing HUK therapy, the overall risk of symptomatic intracranial hemorrhage was 0.67%. Only 1 patient developed hypotensive shock, who was completely relieved with supportive care.^2^

## KLK1 Therapy May Aid Recanalization With Thrombolytics: An Opportunity for Combination Therapy

**Figure 5.**
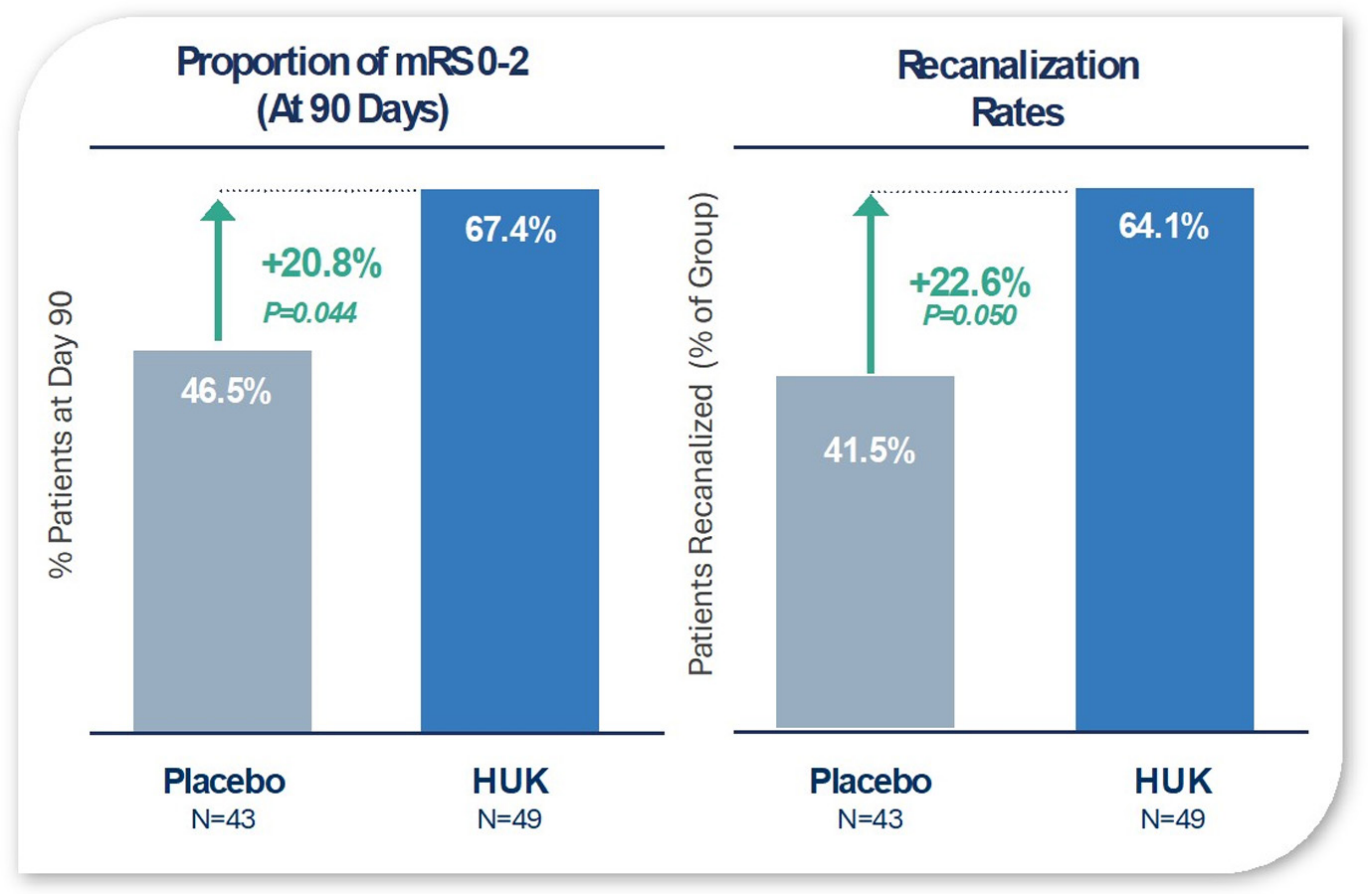
**Treatment with human urinary kallikrein (HUK) after intravenous thrombolysis improves neurological function and reduces stroke recurrence of patients with acute ischemic stroke.** After 14 days of treatment, the HUK group had significantly lower modified Rankin Scale (mRS) scores than the control group (*P*<0.01). and HUK treatment improved the rate of recanalization. HUK also reduced recurrence rates (12.8% vs 21.8% in controls; *P*<0.001).

Preliminary research indicates that KLK1 therapy may enhance the fibrinolytic effects of intravenous thrombolytics (Figure 5).^48–50^ A systematic review and meta-analysis of randomized controlled trials of HUK plus alteplase demonstrated that the combination could improve neurological function recovery after 14 days and the quality of life after 90 days and reduce the adverse reactions of alteplase.^51^ This observation needs confirmation in larger populations. The mechanisms underpinning HUK synergism with alteplase remain speculative. KLK1 does not directly activate plasmin generation.^7^ However, in intact vascular systems, bradykinin was reportedly able to induce the fibrinolytic cascade via alteplase release induction and to inhibit thrombin-induced platelet aggregation.^52,53^ Bradykinin stimulates alteplase release from vascular endothelial cells through a mechanism involving endothelium-derived hyperpolarizing factor and K^+^/Ca channels.^54,55^ Both NO and prostacyclin can be involved in the antiplatelet activities of kinin.

## Initial Evidence Suggests That KLK1 Therapy Reduces the Risk of Stroke Recurrence

As mentioned above, low KLK1 levels are associated with greater risk for stroke recurrence.^14^ Thus, there is potential that an increase of KLK1 could reduce the risk of recurrent strokes. In the RESK study, a single-arm, multicenter, phase IV trial, there was a 2.5% incidence of stroke recurrence after 90 days.^2^ In the ReMEDy1 trial, there was a 2.2% incidence of stroke recurrence after 90 days in patients with AIS taking DM199.^44^ Both compare favorably to the expected recurrence risk of 10% in similar populations.^56,57^ In a retrospective single-center study, there was an 8.9% absolute reduction in the incidence of stroke recurrence after 90 days compared with placebo. In the DM199 ReMEDy1 phase 2 AIS trial, there was a statistically significant 19% reduction in stroke recurrence after 90 days in patients with AIS taking rKLK1 compared with placebo.^44^

Underpinning mechanisms are likely related to a possible action of KLK1 on platelet activation and aggregation. Additionally, KLK1 may reduce the risk of thrombogenesis through re-endothelialization of unstable plaques. Re-endothelialization is a process where resident vascular cells and angiogenic circulating cells recreate an antithrombogenic endothelial layer to heal an ulcerated plaque. The kinin-mediated increase in NO levels can stimulate the proliferation of local endothelial cells and the recruitment of endothelial progenitors expressing the kinin BK2 receptor from circulation.^58^ This may prevent the formation of new thrombi, their embolization, and consequently the occurrence of new strokes. Moreover, a more abundant collateral circulatory system promoted by exogenous KLK1 administration could increase the cerebral flow reserve, thereby limiting the risk of and damage from a new stroke.^59^

## Conclusions

KLK1 augmentation therapy enhances collateral circulation in the ischemic penumbra by generating new bradykinin, which binds to the upregulated BK2 receptors in the ischemic region. This process facilitates localized vasodilation of collateral vessels, thereby increasing blood flow in the penumbra while minimizing untoward effects elsewhere. Continued treatment with KLK1 for 21 days could promote the remodeling of collateral vessels. Imaging studies involving HUK treatment in patients with AIS have shown improved perfusion and better functional outcomes.

rKLK1 possesses all the characteristics of an ideal AIS treatment. For the >80% of patients with AIS who are unable to receive primary revascularization treatments with IVT and EVT, rKLK1 can offer a strong alternative approach to improving cerebral blood flow in penumbra. rKLK1 provides a longer revascularization therapeutic window and a low risk of intracranial hemorrhage. Additionally, KLK1 may offer a synergistic effect with revascularization therapies, which may improve AIS patient outcomes.

## Article Information

### Acknowledgments

The authors thank Kristen Hutchinson of Kristen Hutchinson LLC for medical writing an early draft of this manuscript for which she was funded by DiaMedica Therapeutics Inc. This manuscript was prepared according to the International Society for Medical Publication Professionals’ “Good Publication Practice for Communicating Company-Sponsored Medical Research 2022 Update.”

### Sources of Funding

This study was supported by DiaMedica Therapeutics Inc.

### Disclosures

Dr Kasner reports compensation from W.L. Gore & Associates Inc for end point review committee services; grants from Bayer, DiaMedica, and Medtronic to institution; compensation from Bristol-Myers Squibb, UpToDate, and Medtronic for consulting services; and employment by Perelman School of Medicine, University of Pennsylvania. Dr Bath reports stock options in CoMind; employment by University of Nottingham; compensation from Phagenesis and DiaMedica for consultant services; grants from British Heart Foundation and National Institute for Health and Care Research; and compensation from World Stroke Organization and The Stroke Association for other services. Dr Hill reports compensation from DiaMedica Inc and Brainsgate Ltd for consultant services; employment by University of Calgary; and grants from NoNO Inc, Medtronic, Boehringer Ingelheim, Canadian Institutes of Health Research, and MicroVention Inc. Dr Volpi reports stock options in DiaMedica. Dr Giuffre reports stock options in DiaMedica; stock holdings in DiaMedica; and service as Board of Director for DiaMedica. Dr Masuoka reports employment by DiaMedica. D. Wambeke reports employment by DiaMedica; stock holdings in DiaMedica; stock options in DiaMedica; and service as Chief Business Officer for DiaMedica. Dr Madeddu reports compensation from DiaMedica for consultant services.
